# Interdisciplinary Step-Up Strategy for Infected Pancreatic Walled-Off Necrosis: Sinus Tract Endoscopic Necrosectomy (STEN) Versus Laparoscopic-Assisted Necrosectomy (LAPN)

**DOI:** 10.3390/jcm15103694

**Published:** 2026-05-11

**Authors:** Valerie Kremo, Julia Mühlhäusser, Hanna Plazer, Isabella Fleischmann, Andreas Scheiwiller, Stephan Baumeler, Simon Bütikofer, Martin Bolli, Francesco Mongelli, Jörn-Markus Gass

**Affiliations:** 1 Department of General Surgery, Cantonal Hospital of Lucerne, Spitalstrasse, 6000 Lucerne, Switzerland; 2 Department of Health Sciences and Medicine, University of Lucerne, 6002 Lucerne, Switzerland; 3 Department of Gastroenterology, Cantonal Hospital of Lucerne, Spitalstrasse, 6000 Lucerne, Switzerland; 4 Department of Surgery, Ospedale Regionale di Bellinzona e Valli, EOC, Via Gallino, 6500 Bellinzona, Switzerland; 5 Faculty of Biomedical Sciences, Università della Svizzera Italiana, Via la Santa 1, 6900 Lugano, Switzerland

**Keywords:** acute necrotizing pancreatitis, pancreatic necrosis, drainage, laparoscopy, endoscopy

## Abstract

**Background/Objectives**: Acute infected necrotizing pancreatitis remains associated with substantial morbidity and mortality. The step-up approach combines minimal-invasive drainage with endoscopic transgastric or percutaneous necrosectomy and has been shown to improve outcomes compared with open surgery. Laparoscopic-assisted necrosectomy (LAPN) may be performed in cases of infected walled-off necrosis (WON) following percutaneous drainage and is typically carried out using laparoscopic instrumentation. A newly implemented interdisciplinary approach includes sinus tract endoscopy, guided necrosectomy (STEN), which employs flexible endoscopy through a surgically created sinus tract and offers a less invasive and more targeted alternative to LAPN, providing improved visualization of complex necrotic cavities and facilitating repeatable step-up debridement. This study aimed to assess the introduction of STEN compared with LAPN in the management of infected WON within a step-up approach. **Methods**: A retrospective analysis of patients with infected walled-off necrosis (WON) treated using a step-up approach between 2019 and 2025 was conducted. Patients who underwent CT-guided percutaneous drainage followed by either STEN or LAPN were included. Demographic characteristics and clinical outcomes were collected. The primary endpoint was a composite outcome comprising major complications and 6-month mortality. Secondary outcomes included overall complication rates, need for reinterventions, and length of hospital stay. **Results**: During the study period, 17 patients were included. All patients were managed using a step-up approach: nine underwent STEN and eight underwent LAPN. In the STEN group, six patients (66.7%) met the primary endpoint, all due to major complications, with no mortality observed. In the LAPN group, the primary endpoint occurred in four patients (50.0%), including one death and three major complications. **Conclusions**: Our study showed that both STEN and LAPN were effective in treating infected WON within a step-up approach. STEN and LAPN showed comparable outcomes. However, these findings should be interpreted as exploratory and with caution given the retrospective design and the small sample size of this study. Further studies with larger patient cohorts are warranted to confirm these findings and to better define the role of this technique in the management of infected necrotizing pancreatitis.

## 1. Introduction

Acute necrotizing pancreatitis remains a severe clinical condition associated with substantial morbidity and mortality. Approximately 20% of patients diagnosed with acute pancreatitis develop walled-off necrosis (WON) [1]. About 30% of patients with WON develop an infection in the further course, which is associated with high rates of organ failure as well as increased mortality [2,3]. Despite advances in intensive care and interventional and surgical techniques, the management of infected WON remains a major therapeutic challenge.

Over the last decades, treatment strategies have changed from open necrosectomy towards minimal-invasive approaches [2,4,5]. The step-up approach, combining initial percutaneous or endoscopic drainage with subsequent necrosectomy in patients with no clinical improvement, has been shown to significantly reduce complications and mortality compared with open surgery [2]. Nevertheless, complication rates, mortality as well as hospital costs, remain high [6,7].

Consequently, both minimal-invasive step-up strategies—endoscopic transgastric and percutaneous surgical drainage, followed by necrosectomy—are increasingly common and accepted practice. In the percutaneous surgical step-up procedure, video-assisted retroperitoneal debridement or laparoscopic-assisted pancreatic necrosectomy (LAPN) are retroperitoneal techniques that can be performed following percutaneous drainage in patients with infected WON. In the first, an incision of 50 mm length is used for retroperitoneal debridement avoiding intraperitoneal surgery [5]. LAPN was then developed as a more minimal-invasive technique that is performed using laparoscopic instrumentation and allows effective retroperitoneal debridement through small 12 mm trocar incisions only while limiting surgical tissue trauma. Previous studies have demonstrated the feasibility and safety of LAPN [8].

In recent years, in cases with complex retroperitoneal collections that can be reached only with difficulties, sinus tract endoscopic-guided necrosectomy (STEN) has emerged as an alternative treatment strategy [9,10].

In the STEN procedure, a flexible endoscope is introduced through a surgically created access tract with improved visualization of complex necrotic cavities enabling targeted debridement. Some small case series of STEN have reported encouraging results concerning feasibility, improved visualization of complex necrotic cavities and facilitating repeatable debridement sessions, resulting in clinical success [9,10,11].

Nevertheless, technical approaches vary substantially, beginning with differences in access—ranging from surgically created tracts to stents placed by gastroenterologists—and extending to considerable variability in procedural execution. A comparison between LAPN and STEN may help specify the advantages and limitations of these two minimal-invasive strategies within a percutaneous surgical step-up approach. The aim of the present study was to compare perioperative outcomes, complications, and clinical success in patients with necrotizing pancreatitis and infected WON treated with a step-up approach using either STEN or LAPN.

## 2. Methods

### 2.1. Study Design, Patient Selection and Outcomes

**Figure 1:**
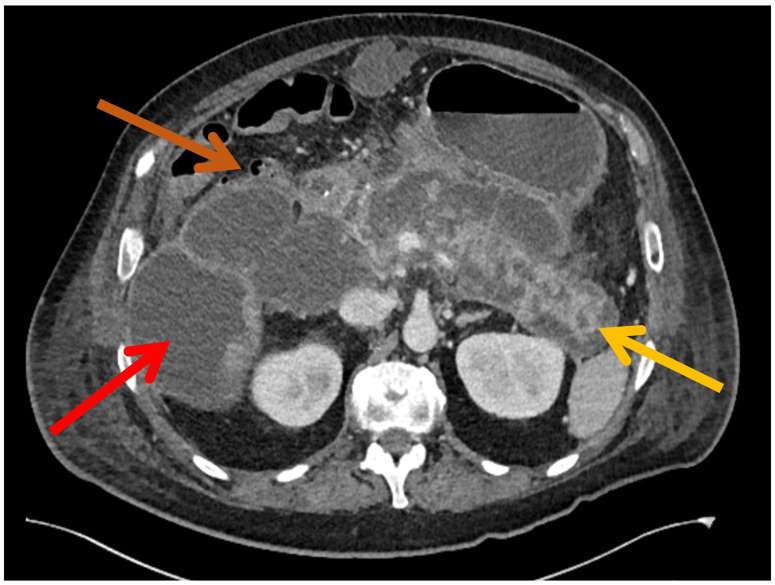
Diagnosis of WON in CT. Red: WON; yellow: pancreas; brown: colon.

This study was designed as a retrospective single-center cohort analysis. At our institution, we reviewed electronic medical records to identify consecutive adult patients (≥18 years) diagnosed with severe acute pancreatitis complicated by infected WON who were treated between 2019 and 2025. In accordance with the established definition, WON was defined as a late-onset, well-demarcated, encapsulated necrosis containing both solid and liquid components occurring at least 4 weeks after the on-set of acute pancreatitis [12]. Diagnosis and follow-up assessment were primarily based on contrast-enhanced cross-sectional imaging (CT), supplemented by magnetic resonance and/or ultrasound as clinically indicated (Figure 1). Infection of the pancreatic collections was diagnosed based on a combination of clinical, radiological, and microbiological criteria, including fever or systemic signs of infection, elevated inflammatory markers, radiological evidence of gas within the collection, marked contrast enhancement of the collection wall or surrounding inflammatory changes, and/or positive blood or drainage fluid cultures [12].

Patients who underwent CT-guided percutaneous drainage followed by either STEN or LAPN were included. Patients with recurrent pancreatitis, pancreatic malignancy, and a history of previous pancreatic or gastric surgery were excluded. As the outcomes of patients treated with a transgastric step-up approach with transgastric drainage followed by transgastric endoscopic necrosectomy if needed and patients treated with percutaneous drainage only have been reported previously by our group [13]; these patients were excluded as well.

Demographic, clinical and procedural data were extracted from institutional databases and included demographic variables (age, sex, body mass index), perioperative risk profile (American Society of Anesthesiology score), etiology of pancreatitis, disease severity at presentation and after 48 h (Ranson score), requirement for intensive care unit admission and duration, antibiotic therapy, and type of intervention performed, including CT-guided percutaneous drainage followed by LAPN or STEN. Post-interventional outcomes included intraoperative and postoperative complications graded according to the Clavien–Dindo classification [14], Comprehensive Complication Index (CCI) [15] and need for reintervention, total hospital length of stay, and discharge destination with assessment of the first 6 months after onset of the present pancreatitis episode. The primary endpoint was a composite outcome including post-interventional major complications, defined as Clavien–Dindo grade III or higher, and all-cause mortality occurring within 6 months from the onset of acute pancreatitis. The primary objective was to compare this composite outcome between patients undergoing STEN and those treated with LAPN. Secondary endpoints comprised overall complication rates, requirement for additional interventions, and total length of hospital stay.

### 2.2. Step-Up Approach and Intervention Techniques

**Figure 2:**
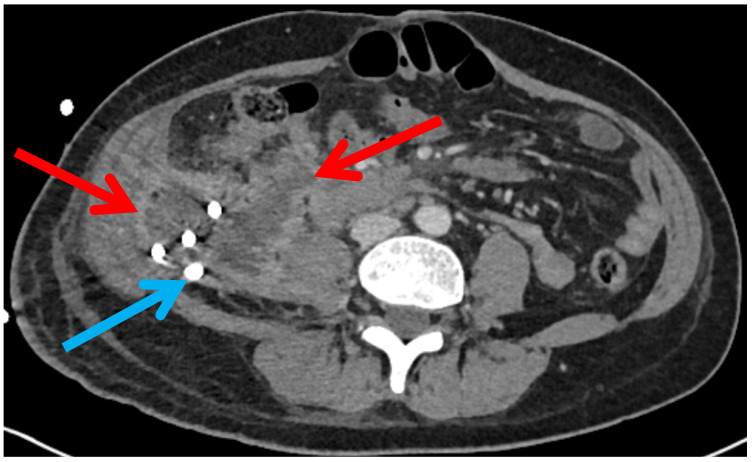
Compact necrotic cavity favoring LAPN over STEN. Red: WON; blue: drainage of the necrotic cavity.

**Figure 3:**
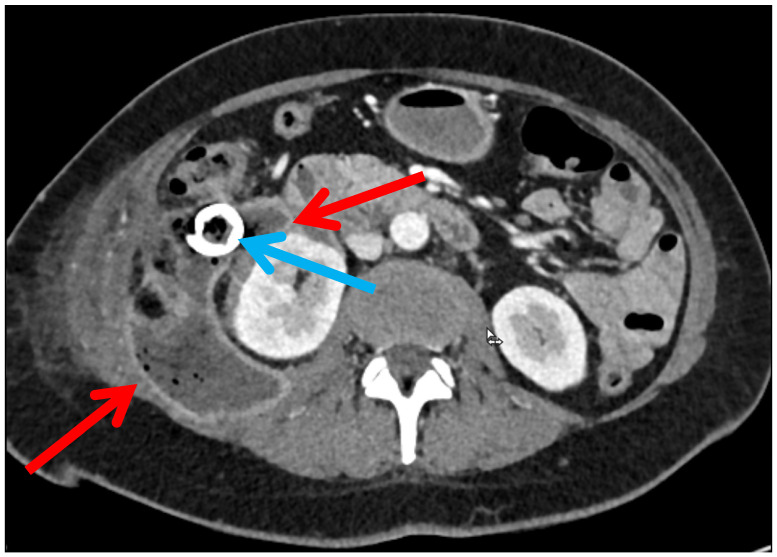
Angulated necrotic cavity favoring STEN over LAPN. Red: WON; blue: ventral drainage of the necrotic cavity.

All patients were managed according to a minimal-invasive percutaneous step-up approach for severe acute pancreatitis. Following CT-guided percutaneous drainage, patients with no clinical improvement requiring a surgical intervention were allocated to one of two groups based on the retroperitoneal necrosectomy technique performed: STEN or LAPN. The indication to proceed with retroperitoneal necrosectomy and the choice between STEN and LAPN were made in cases of suspected infection of a peripancreatic collection, clinical evidence of persistent or recurrent infection despite drainage and antimicrobial therapy and/or insufficient drainage effectiveness (e.g., non-irrigable/dislodged drains, persistent/recurrent cavities) during a multidisciplinary conference. Over the study period, STEN was implemented to address complex retroperitoneal collections that could not be reached by the rigid instruments of LAPN and a progressive shift toward preferential use of STEN was observed. STEN was performed either as primary procedure or secondarily after LAPN in cases of persistent complex cavities. The choice between STEN and LAPN was primarily guided by anatomical accessibility and imaging characteristics on preoperative CT. In particular, anatomical features such as cavity configuration, extent of retroperitoneal involvement, accessibility via the drainage tract, and proximity to surrounding structures were considered during interdisciplinary decision-making. LAPN was selected in patients with less complex or more accessible necrotic cavities, or in the absence of a well-established tract (Figure 2). An advantage of LAPN is the ability to remove larger amounts of necrotic tissue per instrument pass. In contrast, STEN was favored in cases with a complex configuration of the necrotic cavity, particularly in the presence of angulated or branching extensions (Figure 3).

For interpretative purposes, patients were further categorized according to the first retroperitoneal necrosectomy technique performed after percutaneous drainage. “Upfront STEN” was defined as STEN performed as the initial necrosectomy procedure after percutaneous drainage, whereas “upfront LAPN” was defined as LAPN performed as the initial necrosectomy procedure. STEN performed after previous LAPN because of persistent or inaccessible necrosis was considered “salvage STEN after LAPN”. Given the small number of patients, this subgroup classification was used descriptively to clarify treatment sequences and outcome attribution rather than for formal comparative statistical testing.

The decision to pursue a percutaneous step-up strategy with retroperitoneal necrosectomy, rather than a transgastric endoscopic approach, was based on multidisciplinary assessment and considered anatomical characteristics of the WON (e.g., lack of contact with the gastric wall, predominantly solid content), the patient’s clinical condition, and the availability of radiological, endoscopic, and surgical expertise. Intervention was planned as late as possible, preferably at least four weeks after the onset of severe acute pancreatitis.

**Figure 4:**
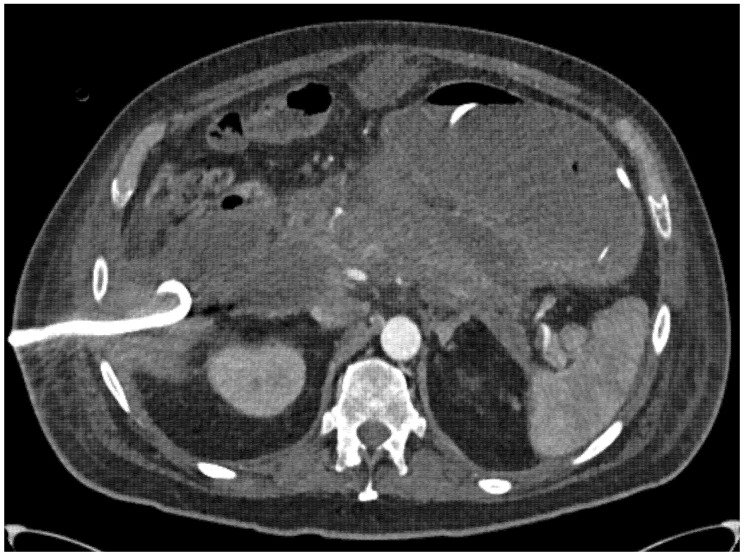
CT-guided drainage of the necrotic cavity.

As part of the surgical step-up pathway, CT-guided percutaneous drainage of the peripancreatic collection was performed as the initial intervention (Figure 4). Drainage placement was jointly planned by interventional radiologists and surgeons to establish a safe and feasible retroperitoneal access route for subsequent necrosectomy, avoiding injury to adjacent organs and the thoracic cavity. Whenever feasible, two pigtail catheters were inserted. In the absence of sufficient clinical improvement following drainage, retroperitoneal necrosectomy was performed using either STEN or LAPN after repeating clinical and imaging reassessment.

**Figure 5:**
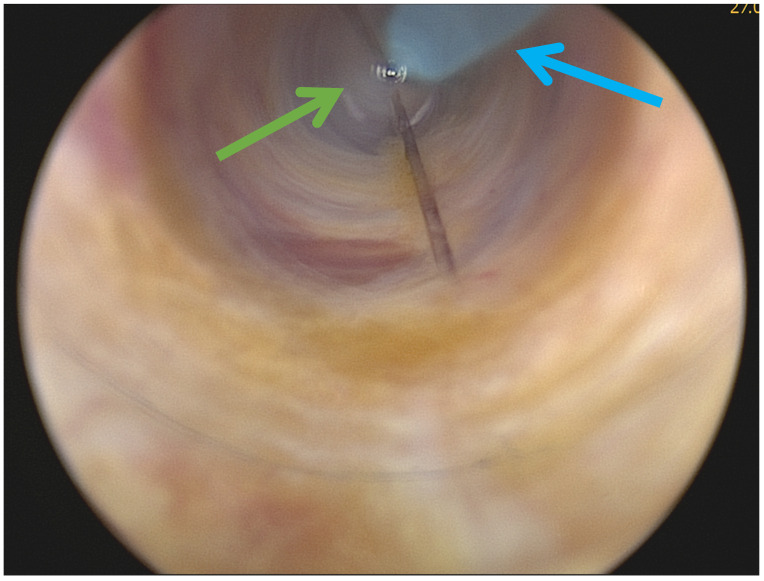
Access to the necrotic cavity through a 12 mm transparent trocar under direct visualization. Green: transparent port; blue: drainage as a guide.

For both techniques, access to the necrotic cavity was obtained through a 12 mm transparent trocar under direct visualization using a 30°-laparoscope along the drain tract into the necrotic cavity by a senior surgeon and a retropneumoperitoneum was created (Figure 5).

All procedures were performed by a consistent interdisciplinary team of experienced radiologists, surgeons and interventional endoscopists, ensuring a high level of procedural standardization.

**Figure 6:**
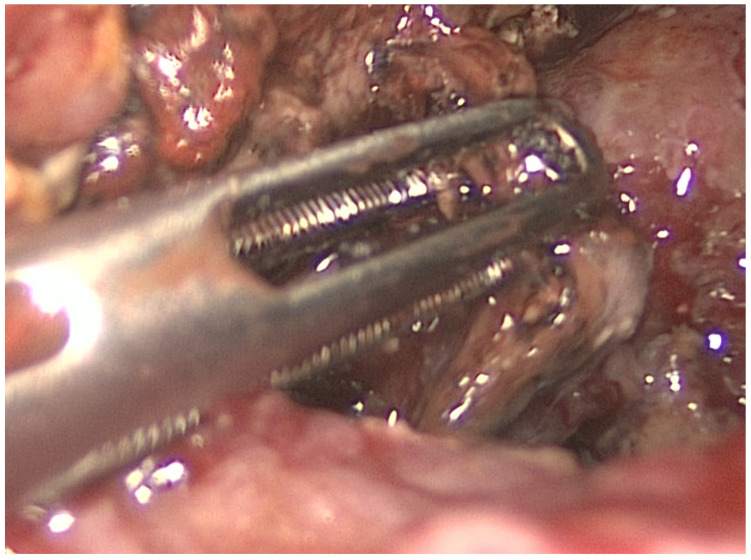
Debridement of necrotic tissue in LAPN.

**Figure 7:**
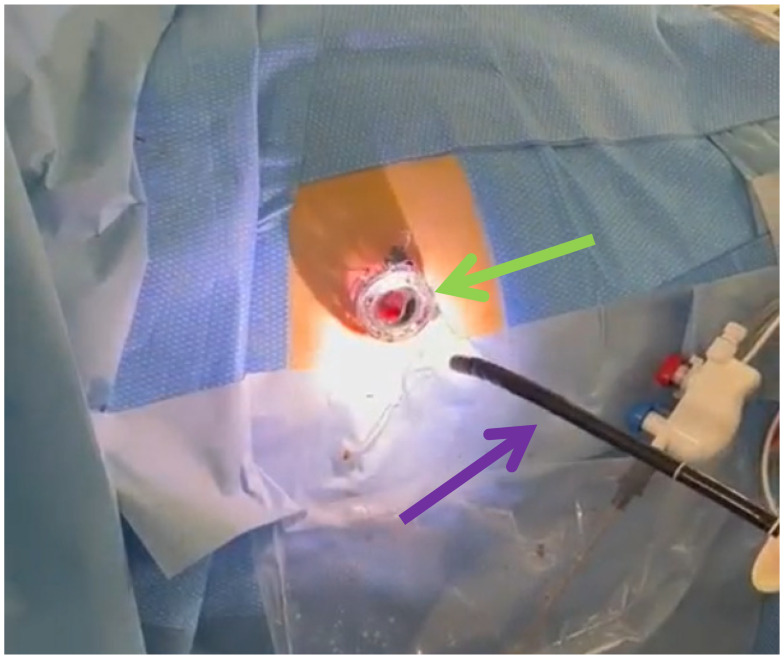
Surgically placed port for introducing a flexible endoscope in STEN. Green: transparent trocar; violet: endoscope.

**Figure 8:**
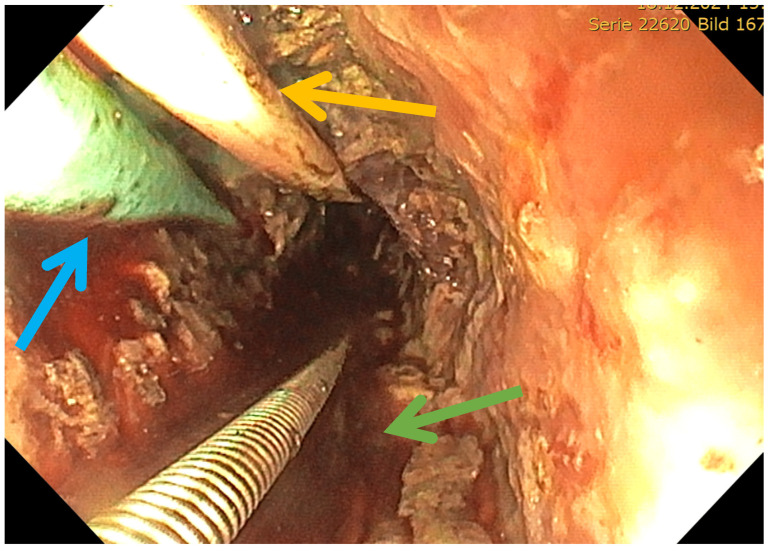
Endoscopic removal of necrotic tissue in STEN. Blue: drain for drainage as guide; yellow: drain for irrigation of the last operation; green: device for endoscopic necrosectomy.

**Figure 9:**
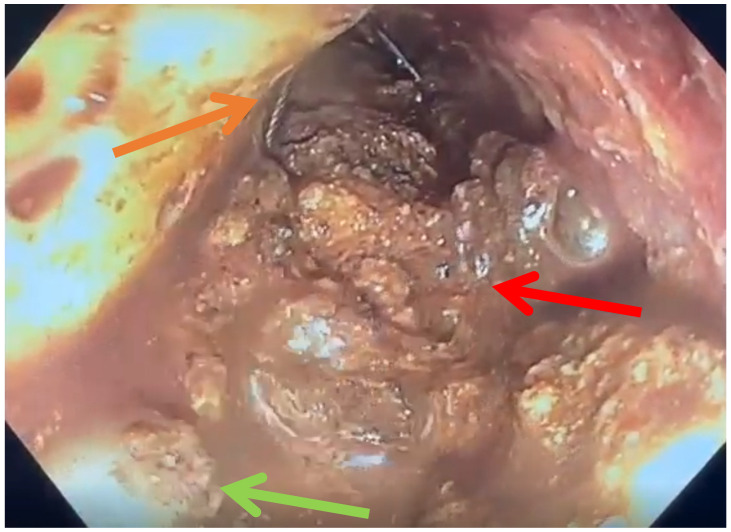
Endoscopic removal of necrotic tissue in STEN. Red: necrotic tissue; green: endoscopic snare; brown: wire of the endoscopic snare.

LAPN was performed by a senior surgeon by placing two to three trocars under direct vision along the previously positioned drainage tract(s). Using a 30° laparoscope and standard laparoscopic instruments, the necrotic material was then debrided and removed, complemented by irrigation of the cavity (Figure 6) [1]. As an alternative, the STEN procedure was introduced within an interdisciplinary treatment pathway. In STEN, a flexible endoscope was advanced through the surgically placed port into the necrotic cavity by a senior gastroenterologist in collaboration with a surgeon (Figure 7). Stepwise removal of devitalized material (e.g., using a snare/retrieval net) was performed under direct visualization with repeated, extensive lavage (Figure 8 and Figure 9). In cases of complex cavity architecture, side cavities/secondary compartments were actively sought and—if safely accessible—opened and debrided. In selected situations, proximity to vascular structures was assessed by endosonography to allow low-risk management of vascular-critical areas or to intentionally limit debridement. STEN allows targeted endoscopic debridement through the established sinus tract and thus provides a potentially even less invasive and more targeted approach. This technique may improve the visualization of complex necrotic cavities and facilitates repeatable step-up debridement sessions.

**Figure 10:**
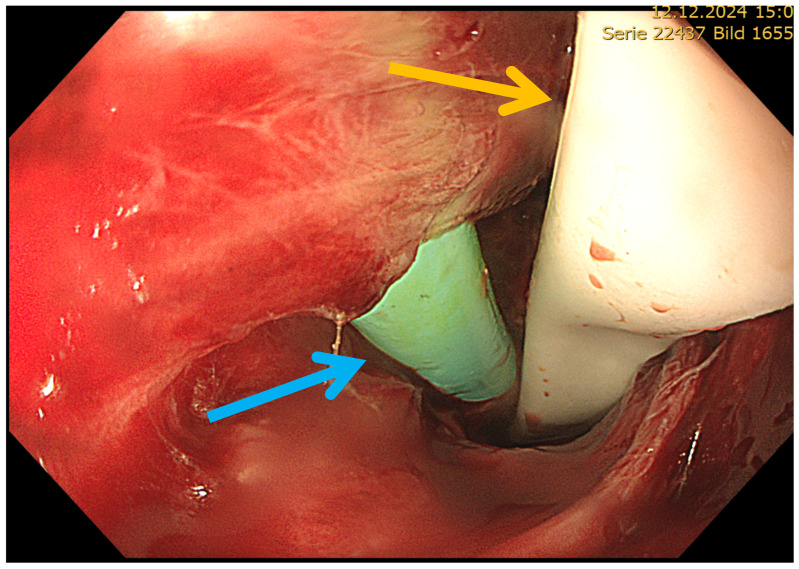
Drainage placement for irrigation and drainage. Blue: drain for drainage as guide; yellow: drain for irrigation.

All procedures were performed under general anesthesia. Intraoperatively, fluid and/or necrotic material were obtained for microbiologic analysis; blood cultures and drainage cultures were collected depending on the clinical course. Antimicrobial therapy was initiated empirically or continued as indicated and subsequently escalated or de-escalated based on pathogen identification and susceptibility testing. At the end of the intervention, drains were left in place, revised, or supplemented for active/passive drainage and irrigation (e.g., repositioning/retaining a pigtail plus additional drains in dependent parts of the cavity) (Figure 10). Postoperatively, a standardized irrigation regimen was applied (intermittent irrigations multiple times daily with defined volumes until effluent was clear, or continuous irrigation over 24 h if required), with outflow via suction/drainage. Irrigability, output volumes, and clinical parameters were documented daily.

Follow-up CT scans were performed at short intervals for re-evaluation (drain position, residual cavities, extent of necrosis); the indication for further necrosectomy was re-established interdisciplinarily based on clinical status, laboratory parameters, drain function, and imaging.

### 2.3. Ethical Statement

The study was approved by the ethics committee EKNZ Switzerland (proposal number 2023-00470). The Strengthening the Reporting of Observational Studies in Epidemiology (STROBE) Statement was followed.

### 2.4. Statistical Analysis

Descriptive statistics were presented as frequencies and percentages for categorical variables. Continuous variables were reported as means with standard deviations (SD) or medians with interquartile ranges (IQR), as appropriate. Given the small sample size, Fisher’s exact test was used for categorical group comparisons when expected cell counts were low; otherwise, the chi-square test was applied. Continuous variables were compared using Student’s *t*-test or the Mann–Whitney U test, according to data distribution. The significance level was set at 0.05. All analyses were conducted using MedCalc^®^ Statistical Software version 23.4.8 (MedCalc Software Ltd., Ostend, Belgium; https://www.medcalc.org; 2026).

## 3. Results

**Figure 11:**
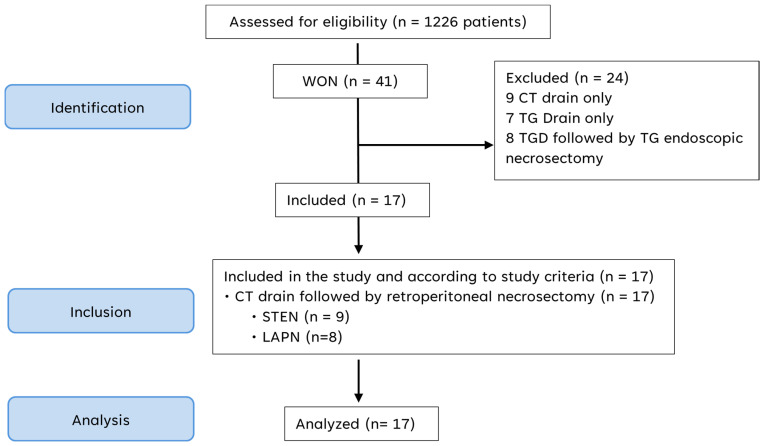
Inclusion of patients. CT: computed tomography. TG: transgastric. TGD: transgastric drain. LAPN: laparoscopic-assisted necrosectomy. STEN: sinus tract endoscopy-guided necrosectomy.

Between 2019 and 2025, a total of 1226 patients were treated for acute pancreatitis at our institution. Among these, 41 patients were identified as having necrotizing pancreatitis complicated by infected peripancreatic collections requiring invasive management. Twenty-four patients were excluded from the present analysis: nine were managed with CT-guided percutaneous drainage alone, seven underwent transgastric drainage only, and eight were treated with transgastric drainage followed by transgastric endoscopic necrosectomy. Outcomes of these treatment strategies have been reported previously by our group [13]. The final study cohort therefore comprised 17 patients, all of whom initially underwent CT-guided percutaneous drainage followed by retroperitoneal necrosectomy. Of these, nine patients were treated with STEN and eight patients underwent LAPN as reported in Figure 11.

The mean patient age was 58.1 ± 14.2 years, and 11 patients (64.7%) were male. The mean body mass index (BMI) was 25.7 ± 4.5 kg/m^2^, and 16 patients (94.1%) were classified with an American Society of Anesthesiology (ASA) score of three or higher. Baseline patient characteristics stratified by treatment group were summarized in Table 1. No statistically significant differences in clinical or demographic variables were observed between the two groups. Post-ERCP pancreatitis was the most common etiology (41.2%), followed by gallstone-related pancreatitis (35.3%). Characteristics of the infected walled-off necrosis were similar between groups and were reported in Table 2.

Microbiological and radiological indicators of infection were comparable between groups (Table 3). Positive blood cultures were observed in two patients (22.2%) in the STEN group and one patient (12.5%) in the LAPN group (*p* = 0.611). Intraoperative microbiological sampling yielded positive results in six patients in each group (66.7% vs. 75.0%, *p* = 0.715). Antibiotic therapy was adapted based on microbiological findings in three patients (33.3%) in the STEN group and five patients (62.5%) in the LAPN group (*p* = 0.243), while concordance between administered antibiotics and identified pathogens was observed in six patients in both groups (66.7% vs. 75.0%, *p* = 0.715). The presence of gas on CT imaging prior to intervention was noted in five patients (55.6%) in the STEN group and three patients (37.5%) in the LAPN group (*p* = 0.470).

Time to first CT-guided drainage was significantly longer in the STEN group (17.0 (IQR 13.2–42.0) vs. 11.0 (IQR 6.0–13.5) days, *p* = 0.030), likely reflecting a progressively less aggressive strategy toward early drainage of WON over time. After placement of percutaneous drainage, the mean time to retroperitoneal necrosectomy was 15.0 (6.7–21.7) days in the STEN group and 21.0 (IQR 11.5–29.5) days in the LAPN group (*p* = 0.268). Operative time was comparable between groups (72 (IQR 60–74) minutes for STEN vs. 71 (IQR 61–92) minutes for LAPN; *p* = 0.499). According to the initial retroperitoneal necrosectomy strategy, five patients underwent upfront STEN after percutaneous drainage, whereas eight patients underwent upfront LAPN. Among the upfront LAPN patients, four subsequently required salvage STEN because of persistent complex necrotic cavities that could not be adequately accessed or cleared with rigid laparoscopic instruments. Therefore, the overall STEN group included both patients treated with upfront STEN and patients receiving salvage STEN after previous LAPN. Because of this treatment overlap, complications, reinterventions, and downstream outcomes in patients who underwent both procedures cannot be attributed exclusively to either STEN or LAPN.

Regarding the primary endpoint, a composite of major complications or death within six months after pancreatitis occurred in 10 patients (58.9%): 6 patients (66.7%) in the STEN group and 4 patients (50.0%) in the LAPN group (*p* = 0.499). The risk difference for the composite endpoint (STEN − LAPN) was +16.7% (95% CI −43.1% to +66.4%). One patient in the LAPN group died, no mortality was observed in the STEN group.

Two intraoperative complications occurred, both in the LAPN group: one intestinal perforation complicated by septic shock and early mortality, and one bleeding event that led to interruption of the procedure without postoperative sequelae. Patients underwent a mean of 3.6 ± 2.8 procedures in the STEN group and 2.6 ± 2.1 procedures in the LAPN group (*p* = 0.461). During the postoperative course, 10 patients (58.9%) experienced complications graded Clavien–Dindo ≥ III; details are reported in Table 4. In the STEN group, a total of nine postoperative complications occurred. Major complications included two Clavien–Dindo grade IV events (septic shock due to colonic fistula due to necrosis and due to catheter-related infection) and four Clavien–Dindo grade III complications (pleural empyema requiring VATS, two paralytic ileus requiring feeding tube, and aspiration pneumonia requiring drainage). Three Clavien–Dindo grade II complications were observed, including anemia, delirium and central venous catheter-related infection. In the LAPN group, eight postoperative complications were recorded, including one Clavien–Dindo grade V event (intestinal perforation complicated by septic shock and early mortality), one Clavien–Dindo grade IV complication (septic shock), two Clavien–Dindo grade III complications (small bowel fistula due to necrosis and pneumothorax), and one Clavien–Dindo grade II complication (pulmonary embolism). Hypokalemia was also observed in two patients as a Clavien–Dindo grade I complication. The mean CCI at discharge, after 3 and 6 months, is reported in Table 5. After 6 months the mean CCI was 41.9 in the STEN group and 39.6 in the LAPN group. CCI values are presented descriptively as medians with interquartile range (IQR) given the limited sample size; formal statistical comparisons were not performed due to insufficient statistical power.

Finally, the length of intensive care unit stay was comparable between groups, with a mean duration of 7.0 (IQR 0–39.0) days in the STEN group and 12.0 (IQR 4.0–20.0) days in the LAPN group (*p* = 0.733). Total hospital length of stay was also similar, averaging 65.0 (IQR 56.2–112.7) days for STEN and 63.0 (50.5–110.0) days for LAPN (*p* = 0.740). At discharge, two of nine patients (22.2%) in the STEN group and five of eight patients (71.4%) in the LAPN group were discharged home (*p* = 0.057).

## 4. Discussion

Our study suggests that, even within a percutaneous step-up approach, the treatment of severe acute pancreatitis complicated by infected walled-off necrosis remains difficult and is associated with a high rate of major complications. Both methods, STEN and LAPN achieved effective source control within an interdisciplinary step-up treatment strategy. No statistically significant differences were observed in major complications, mortality, or length of hospital stay; however, these findings should not be interpreted as evidence of equivalence or true comparability, given the limited sample size and wide confidence intervals. These findings support an individualized, interdisciplinary selection of the necrosectomy technique based on anatomical and clinical factors as well as the locally available resources rather than presumed better outcomes of either procedure. To our knowledge, this study represents one of the first comparative analyses of sinus tract endoscopic necrosectomy (STEN) using a trocar-based retroperitoneal access and laparoscopic-assisted necrosectomy (LAPN) within an interdisciplinary step-up approach. In addition, our study highlights the practical implementation of an interdisciplinary strategy combining surgical access and flexible endoscopy and describes the use of STEN both as an upfront and as a salvage technique in anatomically complex necrotic collections. These aspects add clinically relevant insight to the current literature, in which direct comparisons between different retroperitoneal necrosectomy techniques remain limited.

Both the study by Jagielski et al. and the meta-analysis by Gjeorgjievski et al. describe STEN as a safe and clinically effective treatment approach [16,17]. Nevertheless, the described techniques in the literature that are used to perform STEN vary considerably. These techniques include direct access through a preexisting route following video-assisted retroperitoneal debridement, a wire-guided bougie or balloon dilatation with stent insertion, and the use of a rigid or flexible endoscope and instruments [1,9,10,16,18,19]. Carter et al. describe the advantage of rigid instruments in facilitating the removal of necrotic tissue, whereas complex necrotic cavities may not be adequately accessible with this approach [20]. Wu et al. constated the costs and risks of complications like bleeding of double-lumen catheters are lower than fully covered metal stents [21]. To date, there is a paucity of literature comparing the different access methods to perform STEN, and results are difficult to compare because of the different technical methods. However, data concerning the utilization of a trocar as access for STEN is missing.

In most papers, the method performing STEN is primarily described without direct comparison to alternative approaches.

Our results are consistent in the clinical outcome with those of Palzer et al., who utilized a flexible endoscope without using ballon dilatation or a stent as well but using the access route following video-assisted debridement [22]. In their study with a cohort of 19 patients, STEN was proved to be safe and effective, resulting in only minor complications. The indication for STEN was a treatment failure with persistent necrosis after percutaneous drainage and video-assisted debridement with a 5–6 cm incision. In our study, we performed STEN as an upfront strategy in over half of the patients after percutaneous drainage in cases of necrotic cavities that were difficult to reach with rigid instruments, without LAPN as the first treatment step. STEN served as second-line therapy in only four patients after insufficient clinical improvement following LAPN.

Maurer et al. described the benefits of STEN, including the accessibility of WON through only narrow retroperitoneal routes. However, they also acknowledged the challenge of the unfamiliarity of the equipment to most surgeons [11]. To circumvent this predicament, we initiated a collaboration between surgeons and gastroenterologists to benefit from the skills of each specialist. The trocar was inserted by a surgeon, while the necrosectomy with the flexible endoscope was performed by a gastroenterologist. The study of Maurer et al. provides a comprehensive overview of the various treatment options available, but an analysis of the LAPN technique is missing. Instead, there is a comparison with the less minimally invasive video-assisted retroperitoneal debridement which involves a 5–6 cm incision. In a previous paper, our study group described the advantages of the less invasive LAPN technique over the video-assisted retroperitoneal debridement [13].

In our cohort, no wound infection occurred, neither in the STEN or LAPN group. In the literature there is a risk of wound infection in the technique of video-assisted retroperitoneal debridement as it is less minimally invasive than STEN and LAPN [1,11].

A notable disadvantage of STEN compared to LAPN is the scheduling of necrosectomy procedures, which necessitates the availability of both specialists. In the present study, no pancreatic fistula was observed. However, this complication is described in the literature as a potential complication in both techniques [11,23].

This study has several limitations that should be acknowledged. First, its retrospective single-center design and small sample size substantially limit statistical power and preclude robust multivariable adjustment or propensity-based analyses. The limited number of patients also increases the risk of type II error, meaning that potentially clinically relevant differences between STEN and LAPN may not have reached statistical significance. Therefore, the absence of statistically significant differences should not be interpreted as evidence of equivalence or true comparability between the two techniques. The wide confidence interval around the primary composite outcome further reflects substantial imprecision and limits the ability to draw robust comparative conclusions. Consequently, the present findings should be interpreted as exploratory and hypothesis-generating rather than confirmatory. Second, treatment allocation to STEN or LAPN was not randomized but based on multidisciplinary clinical judgment, anatomical considerations, and imaging characteristics, which may have introduced selection bias and confounding by indication. Therefore, the two groups cannot be considered fully exchangeable. In particular, patients selected for STEN may have differed from those selected for LAPN in terms of anatomical complexity, drainage accessibility, timing of intervention, and overall clinical status. In addition, a detailed and standardized radiological characterization of necrotic collections was not available for all patients. Important anatomical features such as multilocularity, retroperitoneal extension, paracolic gutter involvement, vascular proximity, or ductal disruption were not consistently documented and therefore could not be included in the analysis. These factors likely influenced treatment selection and may have contributed to residual confounding between groups. Another important limitation is that the treatment groups were not fully independent, as four patients underwent STEN as a salvage procedure after previous LAPN. This crossover creates an attribution problem, since complications, reinterventions, and overall clinical outcomes in these patients may reflect the cumulative effect of severe disease, prior LAPN, subsequent STEN, and the overall step-up pathway rather than the effect of one isolated necrosectomy technique. Therefore, comparisons between STEN and LAPN should be interpreted with caution, and the present data are better understood as reflecting treatment sequences within an interdisciplinary step-up strategy rather than two completely independent procedural groups. Similarly, the attribution of complications to a specific intervention is limited, particularly in patients undergoing sequential procedures. In these cases, complications may reflect the cumulative effect of disease severity, prior interventions, and the overall treatment pathway rather than a single technique. This also affects the interpretation of the Comprehensive Complication Index (CCI), which is presented descriptively as medians with interquartile range (IQR) without formal statistical comparison due to the limited sample size and associated lack of statistical power. A formal adjustment of complication rates for disease severity was not feasible because of the limited sample size and the small number of outcome events. Although baseline severity was partially described using the Ranson score, ASA classification, ICU admission, and duration of ICU stay, other relevant markers of disease severity, including CT severity index, baseline organ failure, extent of pancreatic necrosis, and systemic inflammatory burden, were not available in a standardized manner for all patients. Differences in these factors may have influenced the observed complication rates and may have contributed to residual confounding between groups. Therefore, the complication profile should be interpreted cautiously and cannot be attributed solely to the necrosectomy technique. In addition, the progressive shift toward increased use of STEN during the study period may have introduced time-period bias, including possible learning-curve effects and changes in the institutional management of infected WON. The longer time to first drainage observed in the STEN group may therefore reflect an evolving, less aggressive step-up strategy later in the study period rather than a technique-related difference. These factors limit causal interpretation and require cautious interpretation of the observed differences between groups. Finally, the heterogeneous clinical course of severe necrotizing pancreatitis, often requiring multiple interventions and prolonged hospitalization, further limits the attribution of outcomes to a single necrosectomy technique. Despite these limitations, this study provides a detailed real-world comparison of STEN and LAPN within a percutaneous step-up approach for infected walled-off necrosis. The findings offer clinically relevant information on feasibility and complication profiles of both techniques and may support multidisciplinary decision-making in centers managing severe acute pancreatitis.

Future studies should aim to better define the role of different retroperitoneal necrosectomy techniques within the step-up approach. In particular, prospective randomized multi-center studies with standardized radiological characterization of necrotic collections and detailed assessment of anatomical complexity are needed to improve comparability between treatment strategies. Furthermore, the evaluation of interdisciplinary approaches combining surgical and endoscopic expertise may help to optimize patient selection and procedural outcomes. Finally, clearer criteria for selecting STEN versus LAPN, as well as their role as primary or salvage interventions, should be established in order to guide clinical decision-making.

## 5. Conclusions

In this cohort of patients with infected walled-off necrosis managed within a percutaneous step-up approach, both STEN and LAPN were associated with high rates of major complications, reflecting the severity of disease in patients requiring retroperitoneal necrosectomy. No significant differences were observed between techniques in terms of major complications, mortality, length of intensive care unit stay, or overall hospital stay. STEN achieved comparable source control without procedure-related mortality, whereas LAPN was associated with a higher rate of intraoperative complications, including one fatal event. Although limited by the small sample size, these findings suggest that STEN may represent a feasible alternative to standard LAPN in selected patients. These results should be interpreted as exploratory and with caution. Further studies with larger patient cohorts are required to confirm these findings.

## Figures and Tables

**Table 1:** Patients’ characteristics.

	STEN *N* = 9	LAPN *N* = 8	*p*
**Age, years (IQR)**	64.0 (56.7–69.0)	55.0 (40.5–68.0)	0.289
**Sex, female (%)**	4 (44.4)	2 (25.0)	0.620
**Body mass index, kg/m^2^ (SD)**	24.2 (21.0–26.7)	26.3 (23.6–29.6)	0.248
**ASA score**			
2, *n* (%)	1 (11.1)	0	0.365
3, *n* (%)	4 (44.4)	6 (75.0)	
4, *n* (%)	4 (44.4)	2 (25.0)	
**Pancreatitis etiology**			
Gallstones, *n* (%)	4 (44.4)	2 (25.0)	0.234
Post-ERCP, *n* (%)	2 (22.2)	5 (62.5)	
Unknown, *n* (%)	3 (33.3)	1 (12.5)	
**Ranson score**			
On admission, pts (SD)	3.0 (1.7–4.0)	2.5 (1.0–3.5)	0.376
After 48 h, pts (SD)	3.0 (3.0–5.0)	3.0 (2.0–4.0)	0.738
**Intensive care admission, *n* (%)**	6 (66.7)	6 (75.0)	1.000
**Intensive care unit stay, days (SD)**	7.0 (0–39.0)	12.0 (4.0–20.0)	0.733

Dichotomous variables were expressed as numbers with percentages, and continuous variables were expressed as medians with interquartile range (IQR). LAPN: laparoscopic-assisted necrosectomy. STEN: sinus tract endoscopy-guided necrosectomy.

**Table 2:** Characteristics of peripancreatic fluid collections.

	STEN *N* = 9	LAPN *N* = 8	*p*
**Diameter 1, cm (SD)**	9.9 (8.6–11.3)	11.5 (6.8–14.0)	0.736
**Diameter 2, cm (SD)**	5.7 (4.5–6.6)	8.8 (5.4–9.3)	0.083
**Area, cm^2^ (SD)**	35.7 (30.3–58.8)	79.0 (30.4–102.8)	0.236
**Type of collection content**			
Fluid, n (%)	5 (55.6)	5 (62.5)	0.778
Necrosis, n (%)	4 (44.4)	3 (37.5)	

Dichotomous variables were expressed as numbers with percentages, and continuous variables were expressed as medians with interquartile range (IQR). LAPN: laparoscopic-assisted necrosectomy. STEN: sinus tract endoscopy-guided necrosectomy.

**Table 3:** Microbiological and radiological characteristics of infected walled-off necrosis.

	STEN N = 9	LAPN N = 8	*p*
**Positive blood cultures**	2 (22.2)	1 (12.5)	0.611
**Positive intraoperative cultures**	6 (66.7)	6 (75.0)	0.715
**Adaption of antibiotic therapy**	3 (33.3)	5 (62.5)	0.243
**Concordance of antibiotic therapy with culture results**	6 (66.7)	6 (75.0)	0.715
**Gas on CT imaging**	5 (55.6)	3 (37.5)	0.470

Dichotomous variables were expressed as numbers with percentages.

**Table 4:** Postoperative complications (Clavien–Dindo classification).

	STEN N = 9	LAPN N = 8	*p*
**Clavien–Dindo complications**			
CD 0, n (%)	0	1 (12.5)	0.447
CD 1, n (%)	0	2 (25.0)	
CD 2, n (%)	3 (33.3)	1 (12.5)	
CD 3a, n (%)	3 (33.3)	2 (25.0)	
CD 3b, n (%)	1 (11.1)	0	
CD 4a, n (%)	2 (22.2)	0	
CD 4b, n (%)	0	1 (12.5)	
CD 5/death, n (%)	0	1 (12.5)	
intraoperative complications, n (%)	0	2 (25.0)	0.473
postoperative complications (CD 1–5), n (%)	9 (100)	8 (100)	1.000
**major complications (CD 3–4), n (%)**	6 (66.7)	3 (37.5)	0.682
**composite endpoint (major complications and death), n (%)**	6 (66.7)	4 (50)	1.000

Dichotomous variables were expressed as numbers with percentages (%). LAPN: laparoscopic-assisted necrosectomy. STEN: sinus tract endoscopy-guided necrosectomy.

**Table 5:** Postoperative complications (Comprehensive Complication Index).

Mean CCI	STEN	LAPN	*p*	All
**CCI at discharge**	39.5 (27.4–48.9)	42.6 (8.7–53.8)	1.000	42.6 (17.8–48.9)
**CCI after 3 months**	39.5 (27.4–49.5)	43.0 (8.7–53.8)	0.773	42.7 (20.9–49.5)
**CCI after 6 months**	39.5 (27.4–53.8)	43.0 (8.7–56.7)	0.700	42.7 (20.9–53.8)

Dichotomous variables were expressed as numbers with percentages, and continuous variables were expressed as medians with interquartile range (IQR).

## Data Availability

The data presented in this study are available on request from the corresponding author due to restrictions on the availability of these data, which were used under license for the current study and so are not publicly available.
